# GATA factors in human neuroblastoma: distinctive expression patterns in clinical subtypes

**DOI:** 10.1038/sj.bjc.6605276

**Published:** 2009-08-25

**Authors:** V Hoene, M Fischer, A Ivanova, T Wallach, F Berthold, C Dame

**Affiliations:** 1 Department of Neonatology, Charité – Universitätsmedizin Berlin, Augustenburger Platz 1, Berlin D-13353, Germany; 2 Department of Pediatric Oncology and Hematology and Center for Molecular Medicine Cologne (CMMC), University of Cologne, Kerpener Str. 62, Cologne D-50924, Germany

**Keywords:** GATA, neuroblastoma, oncogene, sympathetic nervous system, transcription factor

## Abstract

**Background:**

The aim of this study is to elucidate the expression patterns of GATA transcription factors in neuroblastoma and the developing sympathetic nervous system (SNS).

**Methods:**

GATA-2, -3 and -4 and their cofactor friend-of-GATA (FOG)-2 were investigated in primary neuroblastoma by immunohistochemistry, real-time RT-PCR (*n*=73) and microarray analysis (*n*=251). In addition, *GATA-2, -3* and *FOG-2* expression was determined by northern-blot hybridisation. In the developing murine SNS, Gata-4 and Fog-2 were examined by immunohistochemistry.

**Results:**

Although Gata-2, -3 and Fog-2 are expressed in the developing nervous system, Gata-4 was not detected. In contrast, protein expression of all factors was observed in human neuroblastoma. Northern-blot hybridisation and real-time RT-PCR suggested specific expression patterns of the four genes in primary neuroblastoma, but did not show unequivocal results. In the large cohort examined by microarrays, a significant association of *GATA-2*, *-3* and *FOG-2* expression with low-risk features was observed, whereas *GATA-4* mRNA levels correlated with *MYCN*-amplification.

**Conclusion:**

The transcription factors GATA-2 and -3, which are essential for normal SNS development, and their cofactor FOG-2 are downregulated in aggressive but not in favourable neuroblastoma. In contrast, upregulation of GATA-4 appears to be a common feature of this malignancy and might contribute to neuroblastoma pathogenesis.

GATA transcription factors comprise a family of six zinc-finger proteins regulating cell differentiation and proliferation. Initially, GATA-1, -2 and -3 have been grouped as regulators of haematopoiesis, but analyses of mutant mice with homozygous *Gata-2* or *Gata-3* deletion indicate their essential function also in the development of other organs, particularly the nervous system (Pandolfi *et al*, 1995; Zhou *et al*, 2000). During development, *Gata-2* and *-3* are expressed in different neurons of the brainstem and spinal cord, which are critical for the generation and differentiation of sympathetic neurons (Pandolfi *et al*, 1995; Nardelli *et al*, 1999; Lim *et al*, 2000; Zhou *et al*, 2000; Tsarovina *et al*, 2004). In contrast, GATA-4, -5 and -6 are predominantly implicated in heart and gut development (Laverriere *et al*, 1994; Molkentin, 2000). In the normal brain, Gata-4 expression has only been described in migrating gonadotropin-releasing hormone (GnRH)-secreting neurons (Lawson and Mellon, 1998). However, Gata-4 could not be detected in the adult CNS (Lawson and Mellon, 1998). It has recently been demonstrated that a 5-kb proximal promoter of *Gata-4* can drive reporter gene expression in migratory neural crest cells. Yet, endogenous *Gata-4* has not been detected (Pilon *et al*, 2008). In other reports, Gata-4 expression has been described in some neural crest-derived cardiac progenitors (Tomita *et al*, 2005) and in primitive neural stem cell spheres (Hitoshi *et al*, 2004).

Although primarily considered as important transcriptional regulators during embryogenesis, GATA-2, -3 and -4 have also been associated with tumours. They have been suggested both as tumour suppressors and oncogenes (Ohyashiki *et al*, 1996; Laitinen *et al*, 2000; Akiyama *et al*, 2003; Usary *et al*, 2004; Engelsen *et al*, 2008; Ayala *et al*, 2009; Thurisch *et al*, 2009). Thus, repression or activation of GATA function could be important for cancer biology, and the precise function may depend on cofactors and the specific cellular context.

Friend-of-GATA (FOG) proteins are important cofactors for GATA transcription factors. *In vitro*, all GATA factors can interact with FOG-2 (Cantor and Orkin, 2005). FOG-2 may act as a coactivator or corepressor depending on the cell type and the promoter (Holmes *et al*, 1999; Lu *et al*, 1999; Svensson *et al*, 1999). During development, *Fog-2* is expressed in a variety of tissues including the heart and the brain (Cantor and Orkin, 2005). From E11.5 on, it has also been detected in the ganglia of the peripheral nervous system (Tevosian *et al*, 1999). In an adult, *FOG-2* is predominantly expressed in the heart, the brain and the testis (Holmes *et al*, 1999; Lu *et al*, 1999; Svensson *et al*, 1999; Tevosian *et al*, 1999), and has been described in tumours (Laitinen *et al*, 2000).

Neuroblastoma is the most common extra-cranial solid tumour in childhood. The tumours arise from neural crest cells of the sympathetic nervous system (SNS) and are highly heterogeneous in nature and clinical behaviour. Although younger patients with localised tumours and those with stage 4S disease have an excellent prognosis and often follow spontaneous regression, the outcome of older patients with disseminated disease (stage 4) is still very poor despite intensive multimodal treatment (Maris *et al*, 2007). Although the exact mechanisms leading to the divergent neuroblastoma phenotypes have yet not been characterised, it has been noticed that markers of neuronal differentiation are downregulated in unfavourable tumours (Ohira *et al*, 2003; Nakagawara, 2004; Fischer *et al*, 2006). From these data it has been suggested that de-regulation of normal developmental pathways may contribute to the pathogenesis of the biologically distinct neuroblastoma subtypes.

As GATA transcription factors are involved in both tumourigenesis and SNS development, we aimed at characterising the expression patterns of GATA-2, -3, -4 and FOG-2 in primary human neuroblastoma and in neuronal development of mice to evaluate their possible implication in human neuroblastoma.

## Materials and methods

### Neuroblastoma patients and tissue specimens

Neuroblastoma tissue specimens and RNA samples of 251 patients, who were enrolled in the German Neuroblastoma Trials NB90-NB2004, were obtained from the neuroblastoma tumour repository of the German Competence Net Pediatric Oncology und Hematology. Biopsy specimens were taken before the cytotoxic treatment. Informed consent was obtained from all patients. Age at diagnosis ranged from 0 to 296 months (median 15 months). The median follow-up of patients without fatal event was 6.15 years. Tumour stage was determined according to the criteria of the International Neuroblastoma Staging System (INSS). Stage distribution of the patients is as follows: stage 1, *n*=69; stage 2, *n*=44; stage 3, *n*=40; stage 4, *n*=67; and stage 4S, *n*=31. At diagnosis, 94 patients were <1 year, whereas 157 were older than 1 year. Amplification of the *MYCN* oncogene was analysed by fluorescence *in situ* hybridisation and detected in 33 out of 251 neuroblastomas. Molecular classification according to gene expression profiles determined by oligonucleotide-microarrays (Oberthuer *et al*, 2006) separated the cohort into 82 high-risk and 169 low-risk patients.

### Immunohistochemistry

The expression patterns of Gata-4 and Fog-2 protein were analysed by immunohistochemistry in cryosections from CD1 mice. The day of vaginal plug was defined as E0.5. Approval for animal studies was given by the Institutional Review Board (T 0152/05). Embryos were fixed in 4% PFA (Sigma-Aldrich Chemie, Munich, Germany). Before fixation, embryos older than E15 and pups were additionally perfused under lethal anaesthesia. Tissue was embedded in Cryo-M-Bed (Bright Instrument, Huntingdon, Cambridgeshire, UK) and sectioned at 14 *μ*m. The sections were washed in PBS with 0.1% Triton X-100 (Sigma-Aldrich Chemie), then blocked in PBS containing 0.1% BSA Fraction V (Roth, Karlsruhe, Germany), 10% FBS, 0.1% Triton X-100 and 0.05% sodium azide at room temperature for 1 h and incubated with the primary antibody at 4°C overnight. After three washes, sections were incubated with the secondary antibody for 2 h at room temperature and counterstained with DAPI (0.2 mg ml^−1^ in PBST for 5 min; Roche, Mannheim, Germany). Sections of human neuroblastoma tissue were fixed with 4% PFA for 10 min, then washed, permeabilised and stained.

Primary antibodies: anti-GATA-2 (sc-1235), anti-GATA-3 (sc-1236), anti-GATA-4 (sc-25310, all Santa-Cruz Biotechnology, Santa Cruz, CA, USA; 1 : 200), anti-*β* III Tubulin (TUJ-1) (ab53234, Abcam, Cambridge, UK; 1 : 500) and anti-FOG-2 (sc-10755, Santa-Cruz Biotechnology; 1 : 500). Secondary antibodies: Alexa Fluor 488 donkey anti-goat IgG (A11055, Invitrogen, Karlsruhe, Germany), Alexa Fluor 594 goat anti-mouse IgG2a (A21135, Invitrogen), Alexa Fluor 488 goat anti-mouse IgG1 (A21121, Invitrogen) and Cy3 donkey anti-rabbit IgG (711-165-152, Jackson Immunoresearch Europe, Suffolk, UK) diluted 1 : 500–1 : 5000.

Negative controls were treated in parallel with only a secondary antibody. Microscopic observations were carried out under an epifluorescence microscope (AxioPlan 2 Imaging System, Carl Zeiss, Jena, Germany). Photographs were taken with a digital camera (AxioCAM MRc; Carl Zeiss) and AxioVision 4.2 software (Carl Zeiss).

Northern blot, real-time RT-PCR and western blot were carried out as described in the Supplementary material.

### Microarray analyses

Gene expression analyses were carried out using oligonucleotide-microarrays (Oberthuer *et al*, 2006). Expression profiles were generated in dye-flipped duplicates in dual-colour experiments. In brief, 1 *μ*g of linearly amplified Cy3- and Cy5-labelled cRNA was hybridised together with 1 *μ*g of reverse colour Cy-labelled reference cRNA to a customised 11 kb oligonucleotide-microarray (Agilent Technologies, Waldbronn, Germany). Quality control of raw data was carried out using the software package arrayMagic (Buness *et al*, 2005). After normalisation of the expression profiles using the variance stabilisation algorithm (Huber *et al*, 2002), data from dye-flipped chip pairs were averaged to yield one intensity value for every gene probe of each patient. All microarray data are available at the ArrayExpress database (http://www.ebi.ac.uk/arrayexpress; Accession: E-TABM-38).

### Statistics

Statistics for the microarray analyses were carried out using the nonparametric Mann–Whitney test; *P*-values <0.05 were considered as statistically significant.

## Results

### GATA-4 and FOG-2 as well as GATA-2 and -3 are expressed in human neuroblastoma

**Figure 1:**
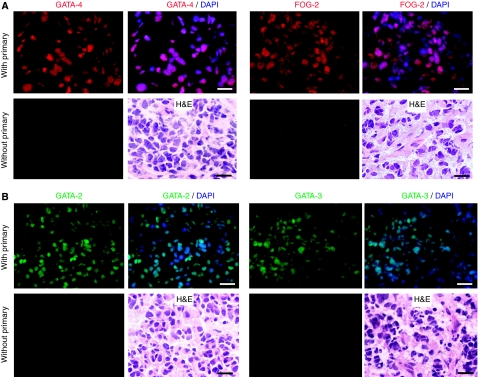
GATA-4 and friend-of-GATA (FOG)-2 as well as GATA-2 and -3 protein detection in human neuroblastoma. (**A**) GATA-4 and FOG-2, plus H&E staining. (**B**) GATA-2 and -3, plus H&E staining. Bars equal 20 *μ*m.

To determine whether the transcription factors GATA-2, -3 and -4 and their cofactor FOG-2 were expressed in human neuroblastoma, expression of these proteins was analysed in primary tumours by immunohistochemistry. We detected GATA-4 and FOG-2 protein (Figure 1A) as well as GATA-2 and -3 protein (Figure 1B) in the cell nuclei. Morphological analysis was performed using H&E staining.

### Gata-4 is expressed neither in the developing and adult brain nor in the developing SNS

**Figure 2:**
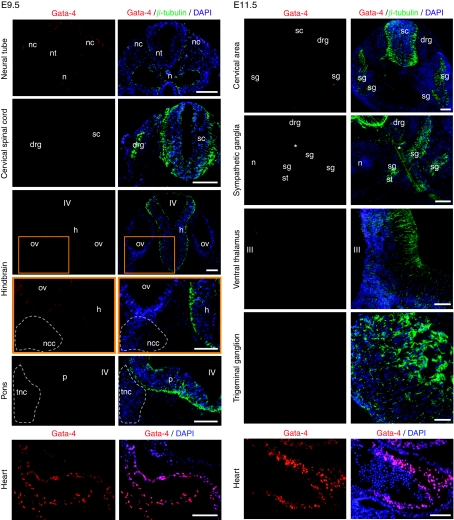
Gata-4 immunohistochemistry in the murine brain, sympathetic nervous system (SNS) and the heart. Left panel from top to bottom: double immunohistochemistry for Gata-4 and *β*-tubulin at E9.5 showing the closing neural tube, the cervical spinal cord, the hindbrain and pons. Right panel from top to bottom: Gata-4 and *β*-tubulin at E11.5 showing the cervical region, sympathetic ganglia, the ventral thalamus and a trigeminal ganglion. The heart served as a positive control. Orange frames represent magnified areas from the lower magnification above. drg: dorsal root ganglion; h: hindbrain; n: notochord; nc: neural crest; ncc: facio-acoustic (VII–VIII) neural crest complex; nt: neural tube; ov: otic vesicle; p: pons; tnc: trigeminal (V) neural crest tissue; sc: spinal cord; sg: sympathetic ganglion; st: sympathetic trunk; III: third ventricle; IV: fourth ventricle; ^*^ventral root. Bars equal 100 *μ*m.

Although the relevance of GATA-2 and -3 for the development of the central and peripheral nervous system has been well characterised (Pandolfi *et al*, 1995; Nardelli *et al*, 1999; Lim *et al*, 2000; Zhou *et al*, 2000; Tsarovina *et al*, 2004), it is not known whether GATA-4 contributes to these processes. We therefore analysed the developing murine brain and SNS for Gata-4 expression using immunohistochemistry. At E9.5 (Figure 2 left panel), there was no Gata-4 in neural crest cells migrating from the closing neural tube, as well as in the dorsal root ganglion. In addition, no Gata-4 was detected in the facio-acoustic and the trigeminal neural crest complexes. The spinal cord, the hindbrain and pons were also Gata-4-negative. At E11.5 (Figure 2 right panel), there was no Gata-4 in the cervical region, including the neural tube, dorsal root ganglia and sympathetic ganglia. Trigeminal ganglia were also negative. There was no labelling in the spinal cord and the thalamus. Sections of the heart (myocardium) served as positive controls and *β*-tubulin for demarcation of areas containing immature neurons. In addition, Gata-4 was undetectable in structures of the SNS at E17.5 and in the brain from E11.5–E18.5, P4.5–P8.5 and adult (not shown).

### Fog-2 is expressed in migrating neural crest cells

**Figure 3:**
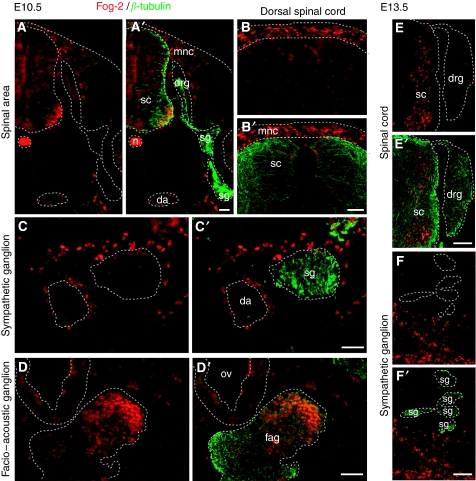
Friend-of-GATA (Fog)-2 protein detection in the neural crest and its derivatives at E10.5 and E13.5. (**A**–**D**) E10.5 and (**E**–**F**) E13.5. Each panel is represented as single and double-stained slide: (**A**–**F**) Fog-2 immunohistochemistry (red), (**A′**–**F′**) double immunohistochemistry of Fog-2 (red) with *β*-tubulin (green) to visualise neuronal projections. (**A**, **A′**) The spinal area. (**B**, **B′**) The dorsal spinal cord with migratory neural crest cells. (**C**, **C′**) The sympathetic ganglion. (**D**, **D′**) The facio-acoustic ganglion. (**E**, **E′**) The spinal cord and dorsal root ganglion. (**F**, **F′**) Sympathetic ganglia. da: dorsal aorta; drg: dorsal root ganglion; fag: facio-acoustic ganglion; mnc: migratory neural crest; n: notochord; ov: otic vesicle; sc: spinal cord; sg: sympathetic ganglion. Bars equal 100 *μ*m.

As FOG-2 is an important cofactor for GATA transcription factors, we were also interested if Fog-2 was expressed in the developing PNS. By immunohistochemistry, we found Fog-2-positive migratory neural crest cells and some post-mitotic neurons in the ventral spinal cord at E10.5 (Figure 3A and B). However, there was no labelling within the dorsal root and sympathetic ganglia (Figure 3A). In addition, Fog-2 was not expressed in ventral sympathetic ganglia (Figure 3C), in contrast to the medial portion of the facio-acoustic ganglion (Figure 3D). Fog-2 staining in migratory neural crest was confirmed at E11.5, as well as the absence of Fog-2 in dorsal root ganglia (data not shown). At E13.5, dorsal root and sympathetic ganglia were also negative (Figure 3E–F).

### Expression levels of *GATA-2*, *-3*, *-4* and *FOG-2* vary in neuroblastoma specimens

To quantify expression levels of *GATA-2*, *-3*, *-4* and *FOG-2* in various subtypes of neuroblastoma, we evaluated their mRNA levels. Using northern-blot analysis, consistent *GATA-2*, *-3* and *FOG-2* expressions were observed in tumours of lower stages (*n*=11), whereas a remarkable reduction in mRNA levels of all three genes was found in neuroblastoma of stage 4 (*n*=3; Supplementary figure S1). Owing to technical limitations in establishing a northern blot for *GATA-4*, expression levels of this gene were determined by real-time RT-PCR in a set of 73 primary tumours (Supplementary table S2). Comparisons of *MYCN*-nonamplified *vs MYCN*-amplified tumours, localised stages *vs* stage 4 *vs* stage 4S and patients below 1 *vs* above 1 year, did not show significantly differing expression levels. Similarly, analysis of *FOG-2*, *GATA-2* and *GATA-3* expression by real-time RT-PCR (Supplementary tables S3–S5) disclosed only lower *FOG-2* levels in *MYCN*-amplified *vs MYCN*-nonamplified tumours at a significant level.

### *GATA-4* is highly expressed in *MYCN*-amplified neuroblastoma

**Figure 4:**
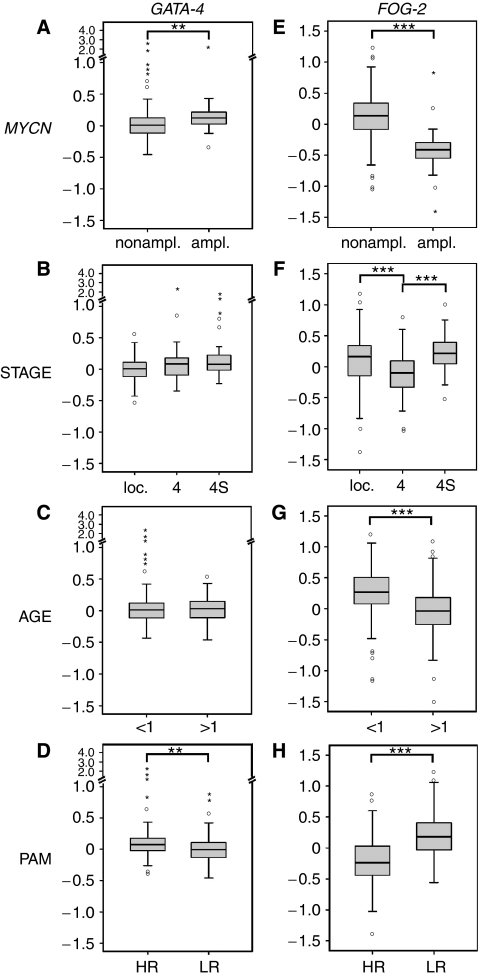
Relative expression levels of *GATA-4* and *friend-of-GATA* (*FOG*)-2 according to microarray analyses. (**A**–**D**) *GATA-4* and (**E**–**H**) *FOG-2*. (**A** and **E**) *MYCN*-nonamplified (*n*=218) *vs MYCN*-amplified (*n*=32 for *GATA-4*; *n*=33 for *FOG-2*). (**B** and **F**) Localised stages (*n*=153) *vs* stage 4 (*n*=67) *vs* stage 4S (*n*=31). (**C** and **G**) Patients below 1 year (*n*=94) *vs* above 1 year (*n*=157). (**D** and **H**) High-risk (HR; *n*=82) *vs* low-risk (LR; *n*=169) tumours according to the PAM classification. Expression values are given in log ratios (sample *vs* reference RNA). Boxes: median expression values (horizontal line) and twenty-fifth and seventy-fifth percentiles; whiskers: distances from the end of the box to the largest and smallest observed values that are <1.5 box lengths from either end of the box; open circles: outlying values; asterisks: extreme values. ^**^P<0.01; ^***^P<0.001 according to the nonparametric Mann–Whitney test.

As the association of *GATA* expression levels and the prognostic phenotype of neuroblastoma could not unequivocally be addressed by northern blot and real-time RT-PCR, we examined *GATA* expression levels by microarray analysis in a larger cohort of primary neuroblastoma (*n*=251). In this set, we compared not only *MYCN*-nonamplified *vs MYCN*-amplified tumours, localised stages *vs* stage 4 *vs* stage 4S, patients below 1 *vs* above 1 year, but also high *vs* low-risk tumours according to a highly accurate gene expression-based classification using the PAM algorithm (Oberthuer *et al*, 2006). *MYCN*-amplified tumours (*n*=32 out of 33; one specimen was not available for *GATA-4* analysis) expressed significantly more *GATA-4* than *MYCN*-nonamplified (*n*=218) tumours (Figure 4A; *P*=0.001). Comparisons of tumours of different stages and from patients of varying ages did not show significant differences (Figure 4B and C), although stage 4 tumours tended to have higher transcript levels than tumours of localised stages. High-risk tumours according to the PAM classification showed significantly higher *GATA-4* expression levels than low-risk tumours (Figure 4D; *P*=0.001).

To further strengthen the significance of GATA-4, protein expression levels of GATA-4 were analysed by western blot. Low- and high-expressing specimens were chosen according to microarray analyses, and protein expression levels were confirmed to correlate well with mRNA expression data (Supplementary figure S2).

### High *FOG-2*, *GATA-2* and *-3* expression levels in neuroblastoma with favourable prognostic markers

*FOG-2* transcript levels determined by microarrays were evaluated for the same 251 tumours. In contrast to *GATA-4*, tumours without *MYCN*-amplification exhibited higher expression values than those with *MYCN*-amplification (Figure 4E; *P*<0.001). Stage as well as age also showed significant differences in *FOG-2* expression. Localised tumours and those of stage 4S had higher transcript levels than stage 4 tumours (Figure 4F; *P*<0.001). Tumours of younger patients (<1 year) expressed more *FOG-2* compared with older patients (>1 year) (Figure 4G; *P*<0.001). Moreover, low-risk tumours according to the PAM classification expressed significantly more *FOG-2* than high-risk neuroblastomas (Figure 4H; *P*<0.001).

**Figure 5:**
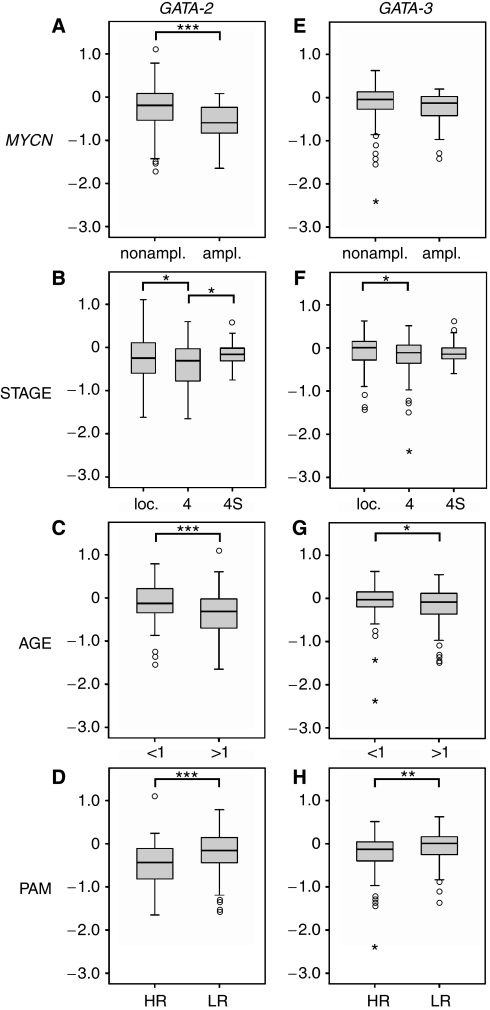
Relative expression levels of *GATA-2* and *-3* according to microarray analyses. (**A**–**D**) *GATA-2* and (**E**–**H**) *GATA-3*. (**A** and **E**) *MYCN*-nonamplified (*n*=218) *vs MYCN*-amplified (*n*=33). (**B** and **F**) Localised stages (*n*=153) *vs* stage 4 (*n*=67) *vs* stage 4S (*n*=31). (**C** and **G**) Patients below 1 year (*n*=94) *vs* above 1 year (*n*=157). (**D** and **H**) High-risk (HR; *n*=82) *vs* low-risk (LR; *n*=169) tumours according to the PAM classification. Expression values are given in log ratios (sample *vs* reference RNA). Box plots represent data as in Figure 4. ^*^P<0.05; ^**^P<0.01; ^***^P<0.001 according to the nonparametric Mann–Whitney test.

As northern blot results suggested differential *GATA-2* and *-3* expression in different clinical neuroblastoma subgroups (Supplementary figure S1), we also analysed their associations with tumour characteristics. Microarray analyses revealed higher transcript levels of both factors in neuroblastoma with favourable prognostic markers, similar to *FOG-2*. *GATA-2* showed significantly higher expression values in *MYCN*-nonamplified compared with *MYCN*-amplified tumours (Figure 5A, *P*<0.001). *GATA-*3 revealed a similar trend, but not a significant association (Figure 5E). Localised neuroblastoma had higher *GATA-2* and *-3* expression levels than tumours of stage 4 (Figure 5B, *P*=0.048) and 5F (*P*=0.039)). Comparisons of stage 4S tumours *vs* stage 4 disclosed a significant association only for *GATA-2* with higher transcript levels in the more favourable 4S tumours (Figure 5B; *P*=0.019). Younger patients had higher *GATA-2* and *-3* expressions than older ones (Figure 5C (*P*<0.001) and 5G (*P*=0.027)). Consistently, the PAM classification indicated significantly higher transcript levels in low-risk tumours for both *GATA-2* and *-3* (Figure 5D (*P*<0.001) and 5H (*P*=0.001)).

Taken together, *GATA-4* expression appears to be a common feature of neuroblastoma with highest expression levels in *MYCN*-amplified tumours. In contrast, *GATA-2*, *-3* and *FOG-2* are preferentially expressed in neuroblastomas with favourable prognostic characteristics.

## Discussion

GATA transcription factors are crucial for the normal development of a variety of tissues. In addition, they have been implicated in the pathogenesis of multiple malignancies. In this study, we characterise the expression patterns of GATA-2, -3, -4 and FOG-2 in murine nervous system development and primary human neuroblastoma to elucidate their potential implication in neuroblastoma pathogenesis. Although GATA-2 and -3 expression has previously been described in various neuroblastoma cell lines (Yang *et al*, 1994; Minegishi *et al*, 2005; Scherzer *et al*, 2008; Wallach *et al*, 2009), we show their expression in primary neuroblastoma for the first time. Furthermore, we demonstrate nuclear GATA-4 protein expression in human neuroblastoma cells. Concerning cofactors for GATA proteins, we also show nuclear FOG-2 expression in primary neuroblastoma. Therefore, FOG-2 is available for interaction with GATA factors.

Moreover, we show that Gata-4 is expressed neither in the developing or adult murine CNS nor in the developing murine SNS, including migratory neural crest. Apparently, GATA-4 expression only arises during tumourigenesis of neuroblastoma, suggesting that it may have a role in the pathogenesis of neuroblastoma. The absence of *Gata-4* in migratory neural crest cells confirms a recent report (Pilon *et al*, 2008), in which whole mount *in situ* hybridisation failed to detect *Gata-4*. Pilon *et al* (2008) discussed the possibility of a small subset of migratory neural crest cells expressing *Gata-4*. In their hands, a 5-kb proximal promoter of *Gata-4* can drive reporter gene expression in migratory neural crest cells at E9.5–E11.5. They discussed that *Gata-4* might only be expressed in a very limited subset of likely cardiac neural crest cells. Tomita *et al* (2005) detected Gata-4 in neural crest-derived cardiac progenitor cells that rest as dormant stem cells in the heart. However, it could be possible that neural crest-derived cardiac progenitors only express Gata-4 when they arrive in the heart. As for primitive neural stem cells, the spheres derived from E6.5 epiblasts have also been reported to express *Gata-4*, in contrast to definitive neurospheres (Hitoshi *et al*, 2004). As epiblasts give rise to all three germ layers, it may not be surprising that *Gata-4* has been detected there. Therefore, we conclude that endogenous Gata-4 is absent in sympathetic structures during mouse development, if not below the detection limit of our method.

We also analysed the expression of Fog-2 in the developing nervous system and confirmed the expression in the facio-acoustic ganglion shown by *in situ* hybridisation at E11.5 (Tevosian *et al*, 1999), but on the protein level and a day earlier in embryonic development. We further specified the staining to the medial portion of the ganglion. Yet, the dorsal root ganglion and sympathetic ganglia were clearly negative. In addition, for the first time we show Fog-2 expression in migratory neural crest cells that may give rise to neuroblastoma. During normal development, FOG-2 might interact with GATA-2 and/or GATA-3, as all three factors are expressed in neural crest derivatives (Tevosian *et al*, 1999; Tsarovina *et al*, 2004).

This allows to conclude that during the development of the nervous system, the four factors investigated show a distinct expression pattern with Gata-4 being unique because of its complete absence. Next, we performed quantitative expression analyses of all four factors in neuroblastoma. Northern blot analysis and real-time RT-PCR of 14 and 73 specimens, respectively, suggested different expression levels in distinct neuroblastoma subgroups of all factors. To evaluate these observations, we used microarray data of 251 primary neuroblastomas, and compared the expression levels of *GATA-2*, *-3*, *-4* and *FOG-2* in different clinico-genetic subgroups. It should be noted that we found significantly higher *GATA-4* expression levels in the unfavourable subgroups of *MYCN*-amplified tumours and of neuroblastoma with unfavourable PAM prediction (Figure 4). This finding is similar to ovarian granulosa cell tumours, where a high GATA-4 expression is associated with aggressive behaviour (Anttonen *et al*, 2005). In mucinous ovarian carcinoma, however, nuclear localisation of GATA-4 correlated negatively with the grade and stage of tumours (Lassus *et al*, 2001). In neuroblastoma, no significant correlation was observed with stage of the disease, although localised tumours tended to have lower *GATA-4* expression than those of disseminated stages 4 and 4S (Figure 4B). In addition, age at diagnosis had no influence on *GATA-4* expression levels. Together, these findings support the notion that GATA-4 is specifically upregulated in neuroblastoma pathogenesis instead of being expressed in progenitor cells of the developing sympathetic system. So far, our microarray analyses suggest higher *GATA-4* expression levels in more aggressive tumours.

It is interesting to note that the GATA cofactor *FOG-2* as well as *GATA-2* and *-3* behave oppositely to *GATA-4*. The expression of *FOG-2* was higher in tumours without *MYCN*-amplification and in tumours with favourable PAM prediction. In contrast to *GATA-4*, *FOG-2* expression did depend on both stage of the tumour and age at diagnosis. Localised tumours and those of stage 4S had higher transcript levels than stage 4 tumours, and tumours of younger patients expressed higher levels of *FOG-2* compared with older patients (Figure 4). In breast cancer, FOG-2 expression is also correlated with favourable prognosis, likely as it is required for the normal expression of its downstream target genes *Esr1* and *Foxa1* (Manuylov *et al*, 2007). Whether FOG-2 is involved in the processes of spontaneous regression in neuroblastoma by supporting cellular differentiation as in breast cancer still remains to be elucidated.

The expression levels of *GATA-2* and *-3* were also higher in the more favourable subtypes of neuroblastoma (Figure 5). Both were more highly expressed in localised tumours (*vs* stage 4), younger patients and tumours with favourable PAM prediction. In addition, significantly higher expression values were also found for *GATA-2* in tumours without *MYCN*-amplification and stage 4S tumours (*vs* stage 4). In an attempt to select genes for prognosis prediction in neuroblastoma, among other genes *GATA-2* has been observed to be associated with favourable prognosis (Ohira *et al*, 2005), which is strengthened by our detailed analysis. In patients with acute myeloid leukaemia, *GATA-2* was rather associated with poor prognosis (Ohyashiki *et al*, 1996), although this correlation is challenged by a recent report (Ayala *et al*, 2009). In breast cancer, high *GATA-3* expression correlates with low tumour grade and slow proliferation rates (Usary *et al*, 2004; Mehra *et al*, 2005), corresponding to our results. In this malignancy, silencing of *GATA-3* is not the primary molecular mechanism that leads to the GATA-3-negative state. Rather, proliferation of GATA-3-negative stem cell-like cells may cause tumour progression (Kouros-Mehr *et al*, 2008). Moreover, using a retroviral delivery strategy Kouros-Mehr *et al* (2008) proved that Gata-3 is sufficient to induce differentiation in breast cancer. This raises the question on upstream regulators of GATA-3 signalling; candidates may be members of the *Wnt* genes (Davidson *et al*, 2002). However, GATA-3 has also been identified as a marker of an aggressive phenotype and poor prognosis in endometrial cancer (Engelsen *et al*, 2008). Thus, GATA factors have been associated with favourable or unfavourable tumours, depending on the type of cancer. In neuroblastoma, favourable tumour subtypes have been described to be more differentiated on the molecular level (Ohira *et al*, 2003; Nakagawara, 2004; Fischer *et al*, 2006). Accordingly, GATA-2 overexpression has been shown to cause differentiation of human neuroblastoma SK-N-BE2 cells (Kaneko *et al*, 2006). In addition, Gata-2 overexpression arrested the proliferation of mouse neuroblastoma cells (NB2a), yet without induction of differentiation (El Wakil *et al*, 2006). In this context, GATA-2 may act on regulators of cell cycle components and/or shut-off the *Notch* pathway (El Wakil *et al*, 2006). Although FOG-2 is likely to interact with any GATA protein, our data obtained in neuroblastoma specimens suggest a predominant interaction with GATA-2 and/or GATA-3; in addition, the interaction between FOG-2 and GATA-4 could be disturbed.

The observation of concordant expression of *Gata-2*, *-3* and Fog-2 in the ganglia of neural crest origin (Tevosian *et al*, 1999; Tsarovina *et al*, 2004), migrating neural crest cells (Fog-2; Figure 3) and favourable neuroblastoma supports the hypothesis that developmental molecular pathways are intact in this subtype of neuroblastoma. Accordingly, the low expression levels of these genes in unfavourable neuroblastoma are in line with a loss of differentiation pathways in these tumours. Moreover, the strong correlation of FOG-2 with favourable markers suggests its possible involvement in the development of a regressive phenotype. In contrast, the finding of GATA-4 expression in primary neuroblastoma, but not in cells of the developing nervous system, indicates its unique role in neuroblastoma pathogenesis.
