# Supplementary material for: GATA factors in human neuroblastoma: distinctive expression patterns in clinical subtypes
Source: Br J Cancer. 2009 Aug 25;101(8):1481–9. doi: 10.1038/sj.bjc.6605276 (PMC2768442; doi:10.1038/sj.bjc.6605276)
Supplement: Supplementary Material [file 6605276x1.doc]

**Supplementary Material**

**Materials and Methods**

# Northern Blot

Ten µg of total RNA from 14 primary neuroblastoma specimens (stage 1: n = 4; stage 2: n = 3; stage 3: n = 3; stage 4: n = 3; stage 4S: n = 1) were size-fractioned on a 1% denaturing formaldehyde agarose gel and transferred onto a nylon membrane (Roche, Mannheim, Germany) using Northern Max One-Hour Transfer Buffer (Ambion, Cambridgeshire, UK). Specific cDNA probes for *GATA-2*, *-3*, *FOG-2* and *β‑actin* were generated by RT-PCR. Primers used for amplification of specific cDNA probes are given in the supplementary Table S1. Blots were hybridized overnight at 42°C in UltraHyb Hybridization Buffer (Ambion) with high sensitivity strippable DNA probes labelled with [α^32^P]-dATP (Strip-EZ DNA Kit, Ambion). After hybridization, membranes were washed, air-dried and exposed to Kodak BioMax MR-1 films (Amersham, Freiburg, Germany).

**Real-time RT-PCR**

Real-time RT-PCR of *GATA-2*, *-3*, *-4* and *FOG-2* was performed using the SYBR Green I reagent on the ABI PRISM 7700 Sequence Detection System (Applied Biosystems, Foster City, CA). PCR reactions were carried out in a total volume of 30 µl containing 26.8 µl SYBR-Green I PCR master mix (Applied Biosystems), 0.4 µl of 1:10 diluted first strand cDNA and 1.4 µl of 2.5 µM forward and reverse primers each (supplementary Table S1). To enable calculation of relative expression levels, serial dilutions of cDNA of the human neuroblastoma cell line IMR-32 were used for generation of standard curves. Cycling conditions comprised a single step at 50°C for 2 min, subsequent 10 min polymerase activation at 95°C, followed by 40 cycles of 95°C for 15 sec and 60°C for 1 min. Data normalization of real-time RT-PCR experiments was performed as described (Fisch*er et* al, 2005). Stage distribution of the 73 patients was as follows: stage 1: n = 12; stage 2: n = 9; stage 3: n = 9; stage 4: n = 34; stage 4S: n = 9. At diagnosis, 29 patients were < 1 year, whereas 44 were older than 1 year. Amplification of the *MYCN* oncogene was detected in 16 out of 73 patients.

**Western Blot**

Protein from neuroblastoma tissue was extracted with RIPA buffer and Western blot analysis was performed with 65 µg protein per lane. The following antibodies were used: anti-GATA-4 (sc-1237, Santa Cruz Biotechnology, Santa Cruz, CA; 1:250) and anti-β-actin (A5441, Sigma, Saint Louis, MO; 1:10000).

**Statistics**

Statistics for the real-time RT-PCR analyses were carried out using the nonparametric Mann-Whitney test; *p* values less than 0.05 were considered as statistically significant.

**Table S1.** Human primer sequences for Northern Blot probes (*β-actin*, *GATA-2*, *GATA‑3*, *FOG-2*) and real-time RT-PCR (*GATA-2*, *GATA-3*, *GATA-4*, *FOG-2*).

| **Primer** | **Sequence** |
| --- | --- |
| *β-actin* FW [NB] | 5’-GATTTAAAAACTGGAACGGTGAAG-3’ |
| *β-actin* RV [NB] | 5’-TAGGATGGCAAGGGACTTCCTG-3’ |
| *GATA-2* FW [NB] | 5’-cagaaccgaccactcatcaagc-3’ |
| *GATA-2* RV [NB] | 5’-agccagggcagctgcactgaag-3’ |
| *GATA-2* FW [PCR] | 5’-GCTGCACAATGTTAACAGGC-3’ |
| *GATA-2* RV [PCR] | 5’-TCTCCTGCATGCACTTTGAC-3’ |
| *GATA-3* FW [NB] | 5’-tgtctgcagccaggagagcag-3’ |
| *GATA-3* RV [NB] | 5’-tggtgtggtccaaaggacagg-3’ |
| *GATA-3* FW [PCR] | 5’-TTAACATCGACGGTCAAGGC-3’ |
| *GATA-3* RV [PCR] | 5’-GGTAGGGATCCATGAAGCAG-3’ |
| *GATA-4* FW [PCR] | 5’-AGGCCTCTTGCAATGCGGA-3’ |
| *GATA-4* RV [PCR] | 5’-CTGGTGGTGGCGTTGCTGG-3’ |
| *FOG-2* FW [NB] | 5’-acgaccctccactgaagaggtctg-3’ |
| *FOG-2* RV [NB] | 5’-gacgtggtcgtccgctcagactg-3’ |
| *FOG-2* FW [PCR] | 5’-GCTTCTATTTTGCCCACAGC-3’ |
| *FOG-2* RV [PCR] | 5’-CTTCTCTTTGCCTCCCACTG-3’ |

**Results**

To quantify expression levels of *GATA-2*, *-3*, *-4* and *FOG-2* in the various subtypes of neuroblastoma, we evaluated their mRNA levels by Northern Blot analysis (*GATA-2*, *-3* and *FOG-2*; Fig. S1) and real-time RT-PCR (Tables S2-5). Northern Blot analysis revealed that *GATA-2*, *-3* and *FOG-2* expression levels were remarkably reduced in stage 4 tumours (highlighted in grey, Fig. S1).

**
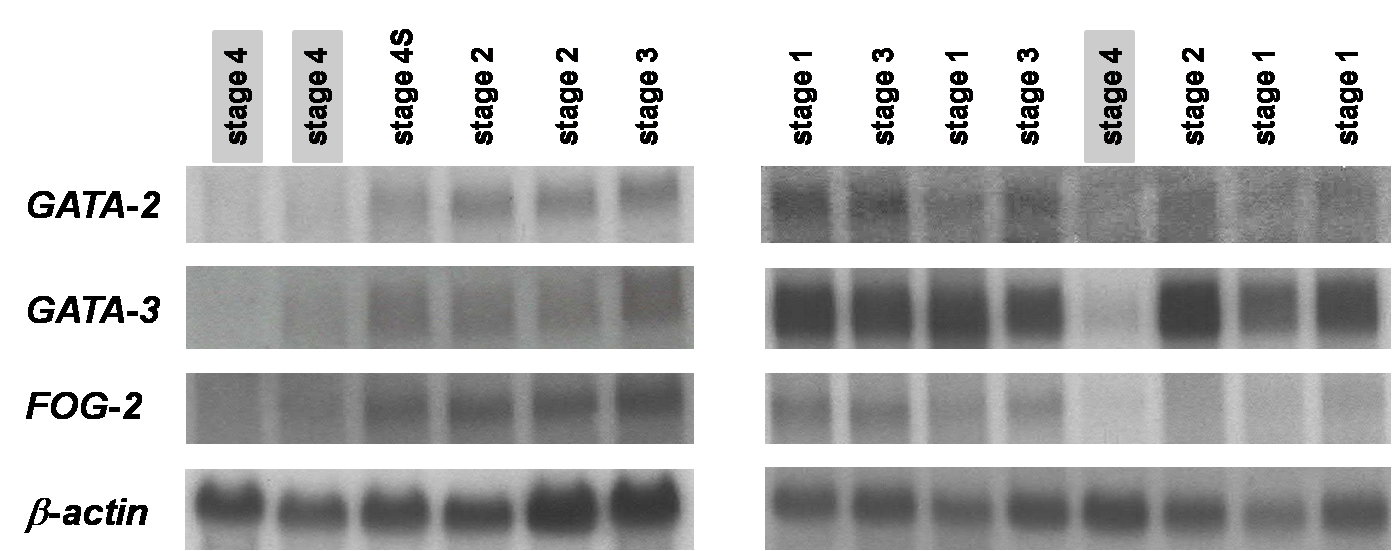
**

**Figure S1.** Northern Blot analysis of *GATA-2*, *-3* and *FOG-2* in 14 primary neuroblastoma specimens.

Using real-time RT-PCR, we observed a significantly lower *FOG-2* mRNA expression in neuroblastoma with *MYCN*-amplification *vs.* *MYCN*-nonamplified neuroblastoma (Table S3). In our main analysis in which we analysed a larger cohort by microarrays (n=251), additional differences in *GATA-2*, *-3*, *-4* and *FOG‑2* expression levels that were associated with *MYCN-*amplification, stage of the neuroblastoma or patients’ age became obvious. Since expression levels correlated well between real-time RT-PCR and microarrays (correlation coefficients ranged from r = 0.53 to r = 0.70), the data obtained by microarray analyses are more robust for statistical analysis due to the higher number of specimens.

**Table S2.** Real-time RT-PCR for *GATA-4* in 73 human neuroblastoma specimens, classified according to *MYCN* amplification, stage and age at diagnosis. Data have been normalized, and the *p* values, median and 25^th^ and 75^th^ percentiles are depicted.

| **Classification** | | ***p* value** | **Median** | **25^th^ p.** | **75^th^ p.** |
| --- | --- | --- | --- | --- | --- |
| MYCN | nonampl. (n = 57) | 0.096 | 1.28 | 0.67 | 1.93 |
|  | ampl. (n = 16) |  | 1.85 | 0.99 | 2.74 |
| Stage | localized (n = 30) | 0.139 (loc. *vs*. 4)  0.338 (4 *vs*. 4S) | 1.36 | 1.22 | 2.04 |
|  | 4 (n = 34) |  | 0.92 | 0.46 | 2.27 |
|  | 4S (n = 9) |  | 1.42 | 0.67 | 11.18 |
| Age | < 1 year (n = 29) | 0.140 | 1.36 | 0.82 | 2.24 |
|  | > 1 year (n = 44) |  | 1.28 | 0.55 | 2.00 |

**Table S3.** Real-time RT-PCR for *FOG-2* in 73 human neuroblastoma specimens. Data representation as in Table S2.

| **Classification** | | ***p* value** | **Median** | **25^th^ p.** | **75^th^ p.** |
| --- | --- | --- | --- | --- | --- |
| MYCN | nonampl. (n = 57) | <0.001 | 5.25 | 2.56 | 7.92 |
|  | ampl. (n = 16) |  | 1.26 | 0.80 | 3.07 |
| Stage | localized (n = 30) | 0.925 (loc. *vs*. 4)  0.199 (4 *vs*. 4S) | 4.37 | 1.81 | 6.48 |
|  | 4 (n = 34) |  | 3.96 | 1.53 | 7.09 |
|  | 4S (n = 9) |  | 6.97 | 4.51 | 8.38 |
| Age | < 1 year (n = 29) | 0.097 | 5.25 | 3.55 | 8.11 |
|  | > 1 year (n = 44) |  | 3.41 | 1.63 | 6.62 |

**Table S4.** Real-time RT-PCR for *GATA-2* in 73 human neuroblastoma specimens. Data representation as in Table S2.

| **Classification** | | ***p* value** | **Median** | **25^th^ p.** | **75^th^ p.** |
| --- | --- | --- | --- | --- | --- |
| MYCN | nonampl. (n = 57) | 0.083 | 25.07 | 10.28 | 43.85 |
|  | ampl. (n = 16) |  | 11.97 | 5.40 | 23.44 |
| Stage | localized (n = 30) | 0.882 (loc. *vs*. 4)  0.511 (4 *vs*. 4S) | 24.13 | 9.22 | 38.38 |
|  | 4 (n = 34) |  | 22.85 | 8.39 | 43.85 |
|  | 4S (n = 9) |  | 14.41 | 0.32 | 33.42 |
| Age | < 1 year (n = 29) | 0.795 | 23.20 | 10.18 | 38.38 |
|  | > 1 year (n = 44) |  | 22.85 | 7.76 | 38.49 |

**Table S5.** Real-time RT-PCR for *GATA-3* in 73 human neuroblastoma specimens. Data representation as in Table S2.

| **Classification** | | ***p* value** | **Median** | **25^th^ p.** | **75^th^ p.** |
| --- | --- | --- | --- | --- | --- |
| MYCN | nonampl. (n = 57) | 0.514 | 1.48 | 1.16 | 1.91 |
|  | ampl. (n = 16) |  | 1.41 | 1.02 | 1.82 |
| Stage | localized (n = 30) | 0.628 (loc. *vs*. 4)  0.420 (4 *vs*. 4S) | 1.42 | 1.06 | 1.88 |
|  | 4 (n = 34) |  | 1.47 | 1.17 | 2.13 |
|  | 4S (n = 9) |  | 1.38 | 1.11 | 1.63 |
| Age | < 1 year (n = 29) | 0.848 | 1.48 | 1.16 | 1.83 |
|  | > 1 year (n = 44) |  | 1.39 | 1.08 | 1.94 |

GATA-4 protein expression levels were analysed by Western Blot. Low- and high-expressing specimens were chosen according to microarray analyses. Indeed, protein expression correlated well with mRNA expression data (Fig. S2).


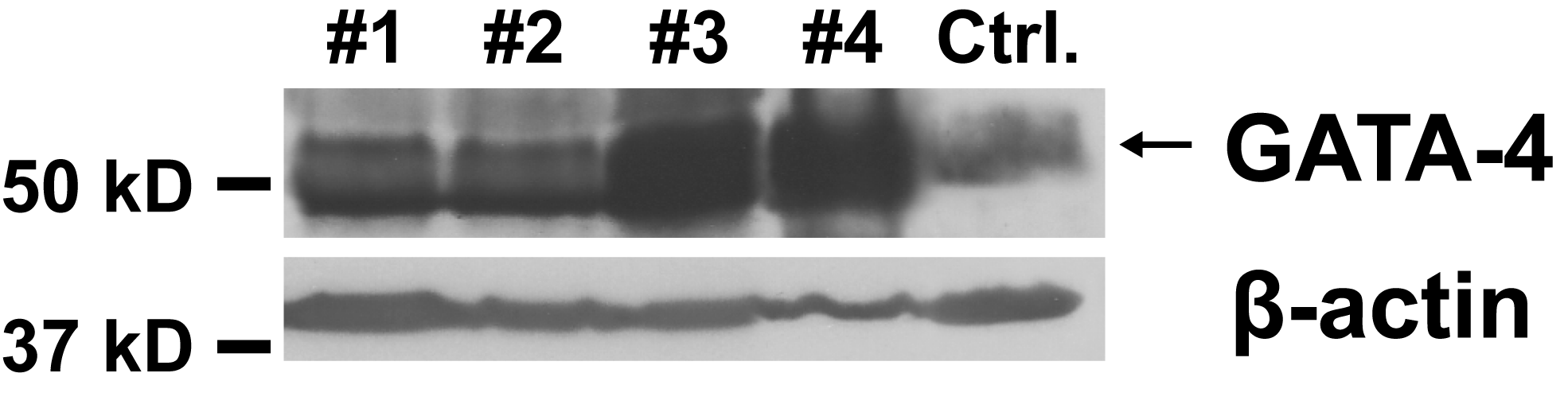


**Figure S2.** Western Blot analysis of four primary neuroblastoma samples and protein from SH-SY5Y neuroblastoma cells as a positive control.

**Reference**

Fischer M, Skowron M, Berthold F (2005) Reliable transcript quantification by real-time reverse transcriptase-polymerase chain reaction in primary neuroblastoma using normalization to averaged expression levels of the control genes HPRT1 and SDHA. *J Mol Diagn* **7:** 89-96
